# Supplementary material for: Comparison of Gastric Alimetry® body surface gastric mapping versus electrogastrography spectral analysis
Source: Sci Rep. 2023 Sep 11;13:14987. doi: 10.1038/s41598-023-41645-w (PMC10495352; doi:10.1038/s41598-023-41645-w)
Supplement: Supplementary file 1 — Supplementary Information. [file 41598_2023_41645_MOESM1_ESM.docx]

Supplementary Figure S1

Average spectrograms for controls (top; n=110), T1D patients (middle; n=32), and NVS patients (bottom; n=43) for BSGM (left) and EGG (right). The lower panel in each shows the median amplitude curve with the 25th-75th percentile shaded.

**
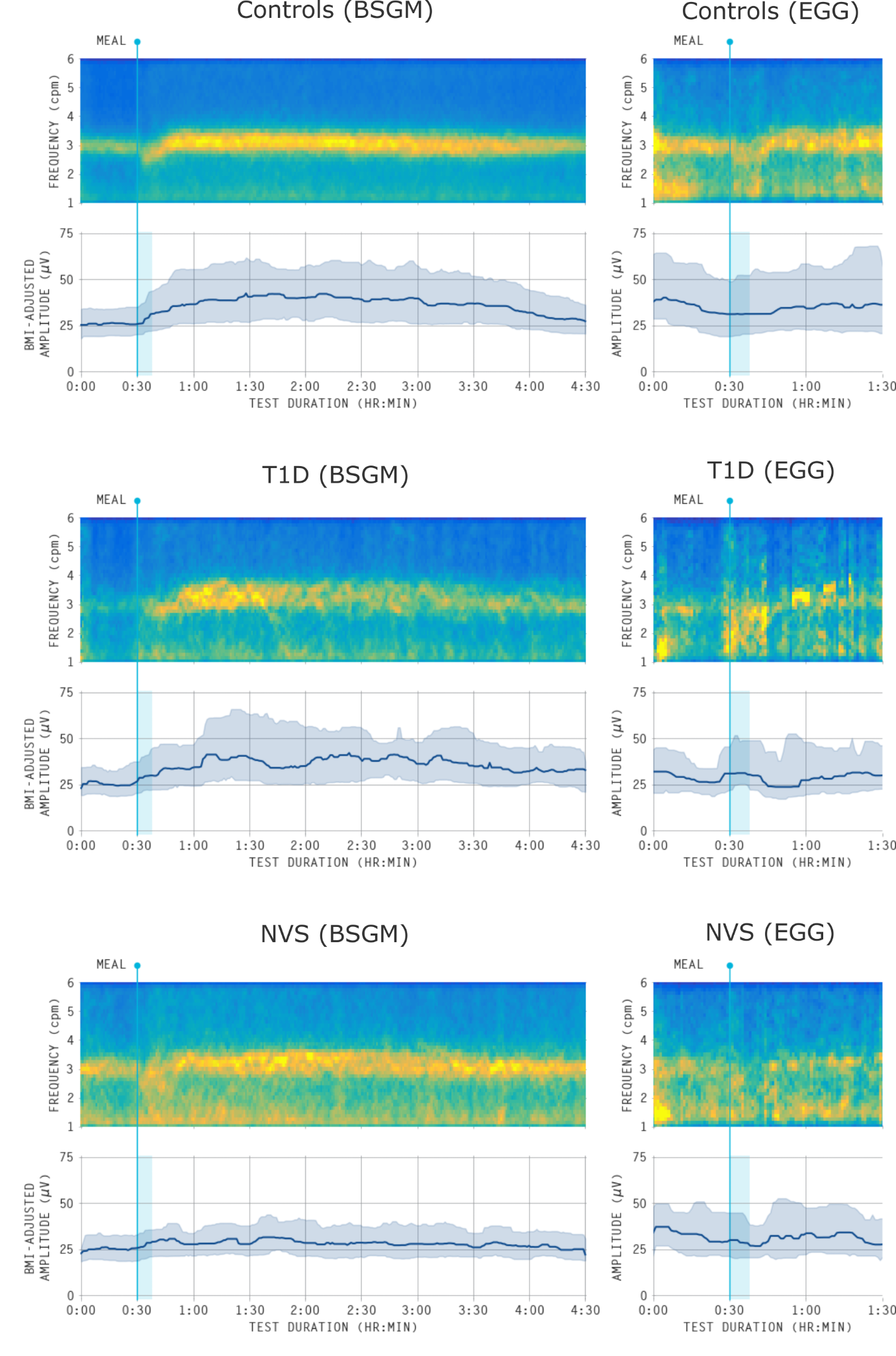
**

|  | Symptom | Preprandial | 0-1hr Postprandial | 1-2hr Postprandial | 2-3hr Postprandial | 3-4hr Postprandial | Overall |
| --- | --- | --- | --- | --- | --- | --- | --- |
| Overall | Bloating | 0.57 (1.48) | 1.01 (2.01) | 0.92 (1.94) | 0.78 (1.84) | 0.67 (1.65) | 0.81 (1.75) |
|  | Heartburn | 0.21 (0.92) | 0.25 (0.95) | 0.24 (0.87) | 0.23 (0.79) | 0.17 (0.56) | 0.22 (0.75) |
|  | Nausea | 0.59 (1.53) | 0.95 (2.09) | 0.88 (1.95) | 0.81 (1.84) | 0.74 (1.73) | 0.81 (1.75) |
|  | Upper Gut Pain | 0.56 (1.51) | 0.76 (1.80) | 0.71 (1.70) | 0.64 (1.59) | 0.60 (1.53) | 0.66 (1.57) |
|  | Stomach Burn | 0.24 (1.03) | 0.38 (1.24) | 0.39 (1.26) | 0.36 (1.19) | 0.34 (1.21) | 0.35 (1.12) |
|  | Excessive Fullness | - | 1.87 (2.71) | 1.20 (2.25) | 0.87 (1.95) | 0.67 (1.75) | 1.16 (2.04) |
|  | Total Symptom Burden | - | - | - | - | - | 5.61 (9.98) |
| Control | Bloating | 0.04 (0.19) | 0.12 (0.34) | 0.05 (0.19) | 0.02 (0.12) | 0.03 (0.16) | 0.06 (0.15) |
|  | Heartburn | 0.03 (0.20) | 0.03 (0.20) | 0.03 (0.20) | 0.02 (0.19) | 0.03 (0.21) | 0.03 (0.19) |
|  | Nausea | 0.02 (0.11) | 0.03 (0.16) | 0.00 (0.04) | 0.00 (0.01) | 0.03 (0.19) | 0.02 (0.06) |
|  | Upper Gut Pain | 0.02 (0.09) | 0.03 (0.14) | 0.00 (0.00) | 0.01 (0.07) | 0.01 (0.13) | 0.01 (0.05) |
|  | Stomach Burn | 0.01 (0.11) | 0.02 (0.11) | 0.01 (0.09) | 0.02 (0.15) | 0.01 (0.13) | 0.02 (0.09) |
|  | Excessive Fullness | - | 0.66 (1.44) | 0.25 (0.83) | 0.14 (0.71) | 0.12 (0.70) | 0.30 (0.83) |
|  | Total Symptom Burden | - | - | - | - | - | 0.84 (1.61) |
| CNVS | Bloating | 1.91 (2.28) | 3.24 (2.62) | 3.11 (2.64) | 2.75 (2.70) | 2.37 (2.56) | 2.74 (2.45) |
|  | Heartburn | 0.60 (1.50) | 0.82 (1.70) | 0.84 (1.56) | 0.77 (1.37) | 0.56 (0.94) | 0.72 (1.31) |
|  | Nausea | 1.99 (2.17) | 3.46 (2.76) | 3.26 (2.60) | 3.04 (2.50) | 2.85 (2.48) | 3.00 (2.25) |
|  | Upper Gut Pain | 1.97 (2.27) | 2.67 (2.55) | 2.63 (2.36) | 2.35 (2.28) | 2.15 (2.27) | 2.40 (2.17) |
|  | Stomach Burn | 0.78 (1.68) | 1.32 (2.07) | 1.37 (2.10) | 1.14 (1.83) | 1.08 (1.86) | 1.16 (1.79) |
|  | Excessive Fullness | - | 4.53 (3.15) | 3.51 (3.08) | 2.61 (2.88) | 2.01 (2.70) | 3.16 (2.79) |
|  | Total Symptom Burden | - | - | - | - | - | 17.89 (12.56) |
| T1DM | Bloating | 0.81 (1.53) | 1.39 (2.10) | 1.34 (1.89) | 1.05 (1.67) | 0.89 (1.36) | 1.11 (1.63) |
|  | Heartburn | 0.40 (1.22) | 0.33 (0.89) | 0.21 (0.60) | 0.28 (0.71) | 0.13 (0.44) | 0.26 (0.69) |
|  | Nausea | 0.75 (1.75) | 0.86 (1.70) | 0.88 (1.47) | 0.89 (1.51) | 0.79 (1.66) | 0.84 (1.43) |
|  | Upper Gut Pain | 0.75 (1.61) | 0.97 (1.77) | 0.84 (1.63) | 0.83 (1.59) | 0.93 (1.74) | 0.87 (1.58) |
|  | Stomach Burn | 0.36 (1.30) | 0.52 (1.41) | 0.48 (1.21) | 0.57 (1.53) | 0.56 (1.58) | 0.50 (1.33) |
|  | Excessive Fullness | - | 2.83 (2.53) | 1.63 (1.78) | 1.23 (1.69) | 0.99 (1.65) | 1.68 (1.71) |
|  | Total Symptom Burden | - | - | - | - | - | 7.24 (8.47) |

**Supplementary Table S1 - Average Symptom Severities**

Mean (standard deviation) symptom severities for each participant subgroup and time window.

|  | | Controls | T1D (v. Controls) | | | NVS (v. Controls) | | |
| --- | --- | --- | --- | --- | --- | --- | --- | --- |
|  |  | Median (IQR) | Median (IQR) | t-statistic | p-value | Median (IQR) | t-statistic | p-value |
| BSGM Metrics | Log BMI-Adjusted Amplitude (μV) | 3.63  (3.38 - 3.93) | 3.56  (3.30 - 3.81) | 0.04 | 0.9685 | 3.38  (3.18 - 3.55) | 3.55 | **0.0005** |
|  | Principal Gastric  Frequency (cpm) | 3.04  (2.90 - 3.18) | 3.15  (2.97 - 3.41) | -3.63 | **0.0004** | 3.09  (2.92 - 3.27) | -1.39 | 0.1678 |
|  | Gastric Alimetry  Rhythm Index | 0.50  (0.39 - 0.64) | 0.43  (0.32 - 0.56) | 2.54 | **0.0121** | 0.30  (0.22 - 0.49) | 5.23 | **<0.0001** |
|  | Log Fed:Fasted  Amplitude Ratio | 0.62  (0.27 - 0.79) | 0.57  (0.35 - 0.81) | -0.27 | 0.7883 | 0.27  (0.09 - 0.53) | 3.61 | **0.0004** |
| EGG Metrics | Log Amplitude (μV) | 3.27  (2.82 - 3.68) | 3.04  (2.88 - 3.49) | 0.56 | 0.5747 | 3.06  (2.90 - 3.38) | 1.56 | 0.1218 |
|  | Dominant Frequency (cpm) | 2.88  (1.50 - 3.12) | 2.50  (1.59 - 3.00) | 1.1 | 0.2752 | 1.62  (1.50 - 2.88) | 3.45 | **0.0007** |
|  | Percentage Time  Normal Frequency | 65.12  (49.05 - 82.22) | 57.85  (40.26 - 67.85) | 2.02 | **0.0453** | 48.44  (36.08 - 61.81) | 3.72 | **0.0003** |
|  | Log Amplitude Ratio | 0.35  (0.10 - 0.66) | 0.34  (0.25 - 0.55) | -1.03 | 0.3065 | 0.09  (-0.06 - 0.36) | 2.96 | **0.0036** |

**Supplementary Table S2 - Group-Level Differences of Spectral Metrics**

Median and interquartile range of each metric for each participant subgroup. Results from independent t-tests are shown for each metric comparing the two patient subgroups with controls. Significant p-values (< 0.05) are shown in bold.

|  | | Preprandial | 0-1hr Postprandial | 1-2hr Postprandial | 2-3hr Postprandial | 3-4hr Postprandial |
| --- | --- | --- | --- | --- | --- | --- |
| Overall | Principal Gastric Frequency (BSGM; cpm) | 2.94  (2.81 - 3.12) | 3.12  (2.97 - 3.28) | 3.11  (2.94 - 3.29) | 3.01  (2.85 - 3.20) | 2.96  (2.84 - 3.14) |
|  | Dominant Frequency (EGG; cpm) | 2.50  (1.50 - 3.00) | 1.88  (1.38 - 3.12) | - | - | - |
|  | BMI-Adjusted Amplitude (BSGM; uV) | 26.28  (20.98 - 33.44) | 35.18  (27.49 - 51.57) | 36.35  (27.70 - 53.48) | 34.52  (26.59 - 48.72) | 31.35  (25.45 - 39.49) |
|  | Amplitude (EGG; uV) | 21.39  (16.75 - 29.56) | 28.92  (21.13 - 46.07) | - | - | - |
|  | Gastric Alimetry Rhythm Index (BSGM) | 0.39  (0.25 - 0.55) | 0.45  (0.24 - 0.64) | 0.51  (0.35 - 0.70) | 0.50  (0.35 - 0.65) | 0.47  (0.29 - 0.63) |
|  | Percentage Time Normal Frequency (EGG; %) | 51.72  (39.29 - 75.00) | 62.59  (36.84 - 94.59) | - | - | - |
| Control | Principal Gastric Frequency (BSGM; cpm) | 2.94  (2.80 - 3.11) | 3.10  (2.97 - 3.24) | 3.07  (2.94 - 3.26) | 2.96  (2.81 - 3.13) | 2.96  (2.83 - 3.10) |
|  | Dominant Frequency (EGG; cpm) | 2.81  (1.53 - 3.00) | 2.69  (1.50 - 3.12) | - | - | - |
|  | BMI-Adjusted Amplitude (BSGM; uV) | 26.58  (20.86 - 32.80) | 37.74  (30.25 - 54.24) | 41.98  (30.51 - 58.04) | 37.59  (29.07 - 54.66) | 30.92  (26.02 - 43.40) |
|  | Amplitude (EGG; uV) | 21.69  (17.58 - 29.74) | 35.47  (23.54 - 53.53) | - | - | - |
|  | Gastric Alimetry Rhythm Index (BSGM) | 0.43  (0.29 - 0.62) | 0.49  (0.31 - 0.69) | 0.55  (0.42 - 0.76) | 0.55  (0.43 - 0.72) | 0.52  (0.36 - 0.68) |
|  | Percentage Time Normal Frequency (EGG; %) | 60.00  (39.39 - 80.00) | 73.43  (44.64 - 100.00) | - | - | - |
| T1DM | Principal Gastric Frequency (BSGM; cpm) | 2.90  (2.81 - 3.28) | 3.21  (3.04 - 3.42) | 3.27  (3.07 - 3.56) | 3.21  (2.98 - 3.50) | 2.98  (2.87 - 3.27) |
|  | Dominant Frequency (EGG; cpm) | 1.88  (1.50 - 2.88) | 1.88  (1.44 - 3.19) | - | - | - |
|  | BMI-Adjusted Amplitude (BSGM; uV) | 25.16  (20.67 - 34.20) | 34.54  (24.94 - 53.24) | 36.19  (28.68 - 51.00) | 40.03  (29.24 - 48.56) | 33.83  (25.94 - 41.56) |
|  | Amplitude (EGG; uV) | 18.43  (15.63 - 29.21) | 28.68  (21.63 - 43.70) | - | - | - |
|  | Gastric Alimetry Rhythm Index (BSGM) | 0.32  (0.21 - 0.45) | 0.46  (0.24 - 0.62) | 0.46  (0.35 - 0.66) | 0.48  (0.37 - 0.64) | 0.43  (0.25 - 0.59) |
|  | Percentage Time Normal Frequency (EGG; %) | 51.61  (40.69 - 59.31) | 61.11  (36.84 - 80.20) | - | - | - |
| CNVS | Principal Gastric Frequency (BSGM; cpm) | 3.01  (2.82 - 3.18) | 3.17  (2.90 - 3.37) | 3.18  (2.95 - 3.44) | 3.05  (2.91 - 3.31) | 3.00  (2.85 - 3.17) |
|  | Dominant Frequency (EGG; cpm) | 1.75  (1.50 - 2.88) | 1.62  (0.31 - 2.75) | - | - | - |
|  | BMI-Adjusted Amplitude (BSGM; uV) | 26.28  (21.09 - 34.00) | 31.00  (24.15 - 35.91) | 29.54  (25.30 - 37.95) | 28.81  (23.33 - 38.88) | 26.66  (23.07 - 33.36) |
|  | Amplitude (EGG; uV) | 22.21  (15.04 - 27.22) | 22.47  (19.69 - 28.69) | - | - | - |
|  | Gastric Alimetry Rhythm Index (BSGM) | 0.37  (0.22 - 0.47) | 0.24  (0.16 - 0.41) | 0.37  (0.22 - 0.57) | 0.39  (0.28 - 0.51) | 0.36  (0.22 - 0.51) |
|  | Percentage Time Normal Frequency (EGG; %) | 46.19  (37.93 - 62.91) | 50.00  (29.03 - 63.64) | - | - | - |

**Supplementary Table S3 - Metrics Calculated for Specific Time Periods**

Median and interquartile range of each metric for each participant subgroup stratified by time.

| Metric | Symptom | Correlation coefficient (95% CI) | p-value | Benjamini-Hochberg Critical Value |
| --- | --- | --- | --- | --- |
| **Gastric Alimetry Rhythm Index** | **Bloating** | **-0.26 (-0.37 - -0.13)** | **0.0005** | **0.0018** |
| **Principal Frequency Deviation** | **Bloating** | **0.22 (0.03 - 0.40)** | **0.0034** | **0.0036** |
| **Gastric Alimetry Rhythm Index** | **Total Symptom Burden** | **-0.21 (-0.34 - -0.08)** | **0.0038** | **0.0054** |
| **Principal Frequency Deviation** | **Excessive Fullness** | **0.21 (0.04 - 0.38)** | **0.0054** | **0.0071** |
| **Gastric Alimetry Rhythm Index** | **Nausea** | **-0.20 (-0.32 - -0.08)** | **0.0062** | **0.0089** |
| **Principal Frequency Deviation** | **Upper Gut Pain** | **0.20 (0.03 - 0.39)** | **0.0074** | **0.0107** |
| **Gastric Alimetry Rhythm Index** | **Upper Gut Pain** | **-0.19 (-0.34 - -0.07)** | **0.0087** | **0.0125** |
| **% Time Normal Frequency** | **Bloating** | **-0.18 (-0.31 - -0.04)** | **0.013** | **0.0143** |
| **Principal Frequency Deviation** | **Total Symptom Burden** | **0.18 (0.03 - 0.35)** | **0.0148** | **0.0161** |
| Gastric Alimetry Rhythm Index | Excessive Fullness | -0.16 (-0.29 - -0.02) | 0.0325 | 0.0179 |
| Dominant Frequency Deviation | Bloating | 0.15 (0.01 - 0.28) | 0.04 | 0.0196 |
| BMI-Adjusted Amplitude | Heartburn | 0.13 (-0.13 - 0.35) | 0.0712 | 0.0214 |
| % Time Normal Frequency | Total Symptom Burden | -0.13 (-0.27 - 0.02) | 0.0718 | 0.0232 |
| Dominant Frequency Deviation | Total Symptom Burden | 0.13 (-0.00 - 0.26) | 0.0787 | 0.025 |
| Principal Frequency Deviation | Nausea | 0.13 (0.00 - 0.28) | 0.0794 | 0.0268 |
| Fed:Fasted Amplitude Ratio | Bloating | -0.13 (-0.26 - 0.06) | 0.0871 | 0.0286 |
| % Time Normal Frequency | Excessive Fullness | -0.12 (-0.27 - 0.03) | 0.0928 | 0.0304 |
| Dominant Frequency Deviation | Nausea | 0.11 (-0.03 - 0.25) | 0.1403 | 0.0321 |
| Dominant Frequency Deviation | Excessive Fullness | 0.11 (-0.03 - 0.24) | 0.152 | 0.0339 |
| Gastric Alimetry Rhythm Index | Stomach Burn | -0.10 (-0.24 - 0.03) | 0.1677 | 0.0357 |
| % Time Normal Frequency | Nausea | -0.10 (-0.24 - 0.04) | 0.1891 | 0.0375 |
| Dominant Frequency Deviation | Upper Gut Pain | 0.10 (-0.05 - 0.24) | 0.1902 | 0.0393 |
| Fed:Fasted Amplitude Ratio | Total Symptom Burden | -0.09 (-0.25 - 0.09) | 0.215 | 0.0411 |
| BMI-Adjusted Amplitude | Nausea | -0.08 (-0.22 - 0.07) | 0.2704 | 0.0429 |
| % Time Normal Frequency | Upper Gut Pain | -0.08 (-0.22 - 0.07) | 0.2864 | 0.0446 |
| Fed:Fasted Amplitude Ratio | Nausea | -0.08 (-0.25 - 0.09) | 0.2995 | 0.0464 |
| Amplitude | Bloating | -0.08 (-0.17 - 0.05) | 0.3072 | 0.0482 |
| Fed:Fasted Amplitude Ratio | Upper Gut Pain | -0.07 (-0.26 - 0.15) | 0.313 | 0.05 |
| Dominant Frequency Deviation | Heartburn | 0.07 (-0.07 - 0.24) | 0.3464 | 0.0518 |
| % Time Normal Frequency | Stomach Burn | -0.07 (-0.24 - 0.11) | 0.3506 | 0.0536 |
| Dominant Frequency Deviation | Stomach Burn | 0.07 (-0.07 - 0.21) | 0.3517 | 0.0554 |
| Amplitude Ratio | Bloating | -0.07 (-0.23 - 0.12) | 0.3784 | 0.0571 |
| Gastric Alimetry Rhythm Index | Heartburn | -0.06 (-0.24 - 0.08) | 0.3954 | 0.0589 |
| Amplitude Ratio | Stomach Burn | 0.06 (-0.16 - 0.35) | 0.4253 | 0.0607 |
| Amplitude Ratio | Total Symptom Burden | -0.05 (-0.24 - 0.15) | 0.4601 | 0.0625 |
| Amplitude Ratio | Heartburn | 0.05 (-0.11 - 0.23) | 0.4721 | 0.0643 |
| BMI-Adjusted Amplitude | Bloating | -0.05 (-0.19 - 0.10) | 0.5069 | 0.0661 |
| Fed:Fasted Amplitude Ratio | Excessive Fullness | -0.05 (-0.20 - 0.13) | 0.5181 | 0.0679 |
| Principal Frequency Deviation | Heartburn | -0.04 (-0.14 - 0.09) | 0.5919 | 0.0696 |
| BMI-Adjusted Amplitude | Total Symptom Burden | -0.04 (-0.19 - 0.16) | 0.6089 | 0.0714 |
| Amplitude | Heartburn | 0.04 (-0.08 - 0.19) | 0.6317 | 0.0732 |
| Fed:Fasted Amplitude Ratio | Stomach Burn | 0.03 (-0.18 - 0.27) | 0.6615 | 0.075 |
| Principal Frequency Deviation | Stomach Burn | 0.03 (-0.12 - 0.18) | 0.6719 | 0.0768 |
| Fed:Fasted Amplitude Ratio | Heartburn | 0.03 (-0.14 - 0.21) | 0.7308 | 0.0786 |
| Amplitude | Stomach Burn | 0.02 (-0.11 - 0.19) | 0.7397 | 0.0804 |
| Amplitude Ratio | Upper Gut Pain | -0.02 (-0.24 - 0.24) | 0.7499 | 0.0821 |
| Amplitude Ratio | Nausea | -0.02 (-0.22 - 0.17) | 0.7612 | 0.0839 |
| % Time Normal Frequency | Heartburn | -0.02 (-0.19 - 0.13) | 0.8114 | 0.0857 |
| Amplitude | Total Symptom Burden | -0.02 (-0.13 - 0.12) | 0.8179 | 0.0875 |
| Amplitude | Excessive Fullness | 0.02 (-0.11 - 0.17) | 0.8359 | 0.0893 |
| Amplitude Ratio | Excessive Fullness | -0.01 (-0.20 - 0.19) | 0.8442 | 0.0911 |
| BMI-Adjusted Amplitude | Upper Gut Pain | -0.01 (-0.20 - 0.17) | 0.8557 | 0.0929 |
| BMI-Adjusted Amplitude | Stomach Burn | 0.01 (-0.19 - 0.23) | 0.8734 | 0.0946 |
| Amplitude | Nausea | -0.01 (-0.13 - 0.12) | 0.8749 | 0.0964 |
| Amplitude | Upper Gut Pain | -0.01 (-0.13 - 0.14) | 0.9035 | 0.0982 |
| BMI-Adjusted Amplitude | Excessive Fullness | -0.01 (-0.15 - 0.16) | 0.9203 | 0.1 |

**Supplementary Table S4 - Correlation of Spectral Metrics with Symptoms**

Complete results of the metric/symptom correlation analysis. For each metric/symptom pair, we reported the Pearson correlation coefficient with 95% confidence intervals computed using a bootstrapping procedure with 1000 bootstrap samples, the associated p-value, and Benjamini-Hochberg critical values. Data is ordered by p-value and bolded for rows that are significant according to the Benjamini-Hochberg procedure
